# Room Temperature Nanographene Production via CO_2_ Electrochemical Reduction on the Electrodeposited Bi on Sn Substrate

**DOI:** 10.3390/nano12193389

**Published:** 2022-09-28

**Authors:** Piriya Pinthong, Sarita Phupaichitkun, Suthasinee Watmanee, Rungkiat Nganglumpoon, Duangamol N. Tungasmita, Sukkaneste Tungasmita, Yuttanant Boonyongmaneerat, Nadtinan Promphet, Nadnudda Rodthongkum, Joongjai Panpranot

**Affiliations:** 1 Center of Excellence on Catalysis and Catalytic Reaction Engineering, Department of Chemical Engineering, Faculty of Engineering, Chulalongkorn University, Bangkok 10330, Thailand; 2 Graphene Electronics Research Unit, Faculty of Science, Chulalongkorn University, Bangkok 10330, Thailand; 3 Department of Chemistry, Faculty of Science, Chulalongkorn University, Bangkok 10330, Thailand; 4 Department of Physics, Faculty of Science, Chulalongkorn University, Bangkok 10330, Thailand; 5 Metallurgy and Materials Science Research Institute (MMRI), Chulalongkorn University, Bangkok 10330, Thailand; 6 Bio-Circular-Green-economy Technology & Engineering Center (BCGeTEC), Department of Chemical Engineering, Faculty of Engineering, Chulalongkorn University, Bangkok 10330, Thailand

**Keywords:** electrochemical reduction of CO_2_, Bi/Sn electrode, graphene

## Abstract

Electrochemical reduction of carbon dioxide (CO_2_RR) to crystalline solid carbon at room temperature is challenging, but it is a providential CO_2_ utilization route due to its indefinite storage and potential applications of its products in many advanced technologies. Here, room-temperature synthesis of polycrystalline nanographene was achieved by CO_2_RR over the electrodeposited Bi on Sn substrate prepared with various bismuth concentrations (0.01 M, 0.05 M, and 0.1 M). The solid carbon products were solely produced on all the prepared electrodes at the applied potential −1.1 V vs. Ag/AgCl and were characterized as polycrystalline nanographene with an average domain size of ca. 3–4 nm. The morphology of the electrodeposited Bi/Sn electrocatalysts did not have much effect on the final structure of the solid carbon products formed but rather affected the CO_2_ electroreduction activity. The optimized negative potential for the formation of nanographene products on the 0.05Bi/Sn was ca. −1.5 V vs. Ag/AgCl. Increasing the negative value of the applied potential accelerated the agglomeration of the highly reactive nascent Bi clusters in situ formed under the reaction conditions, which, as a consequence, resulted in a slight deviation of the product selectivity toward gaseous CO and H_2_ evolution reaction. The Bi–graphene composites produced by this method show high potential as an additive for working electrode modification in electrochemical sensor-related applications.

## 1. Introduction

The global climate change problem is becoming more serious since the accumulation of carbon dioxide (CO_2_) and other long-lived greenhouse gases in Earth’s atmosphere have continuously increased. Production of valuable products from carbon dioxide (CO_2_) is a challenging task. Among the various CO_2_ conversion processes, electrochemical reduction of CO_2_ (CO_2_RR) has been an interesting approach due to mild operating conditions, high energy efficiency, and the possibility to use renewable energy sources. Various gaseous, liquid, and solid products can be produced from CO_2_RR, such as formate/formic, carbon monoxide, alcohol, hydrocarbons, and amorphous/crystalline solid carbon. Solid carbon products are of particular interest because of their indefinite storage aspect and their potential applications in many advanced technologies. However, CO_2_RR-to-solid-carbon products is an emerging phenomenon that has not been widely explored.

Typically, under CO_2_RR systems, carbon deposits can be formed on an electrode surface after a long reaction time, causing catalyst deactivation [1]. However, there have been a number of new cases showing that solid carbon is produced as the major CO_2_RR products [2,3,4,5]. CO_2_RR-to-amorphous-carbon products were reported to occur on ceria-containing electrocatalysts in a liquid metal system [2]. The reduction of Ce^3+^ to Ce^0^ was suggested to play an important role to convert CO_2_ to a carbon product. The obtained product has been described as amorphous carbonaceous nanosheets with a typical thickness of 3 nm. In a more recent study by our group [3], the production of 3D-nanostructured carbon allotropes (~1 μm thick) from CO_2_ was achieved on various metal electrocatalysts including silver, zinc, cobalt, and bismuth at room temperature in a ternary electrolyte system containing propylene carbonate (PC), 1-butyl-3-methyl-imidazolium-tetrafluoroborate ([BMIM]BF_4_) and water at the applied potential ranging from −1.1 V to −1.6 V vs. Ag/AgCl [3]. The formation and growth characteristics of the CO_2_RR-derived nanostructured carbon films have been elucidated on the nascent Ag clusters formed under room-temperature CO_2_RR [4]. The CO_2_RR-to-solid-carbon on bismuth electrode has been studied to a lesser extent. 

Bismuth has been an attractive metal electrocatalyst for CO_2_RR due to its low price, long-term stability, low environmental impact, and high selectivity for CO production [6,7]. The electrodeposition method is widely adopted for preparation of bismuth-based electrocatalysts with controllable morphology that can improve their electrocatalytic performances in CO_2_RR [8]. For example, Koh et al. [9] produced hierarchical Bi dendritic catalysts by electrodeposition with a formate faradaic efficiency of ~89% at −0.74 V vs. RHE and stable performance over a 12 h operation. Qiu et al. [10] created a series of size-tunable nanobismuth-based catalysts for the CO_2_RR to formate. They discovered that the size significantly affected the electrochemically active surface area, current density, and faradaic efficiency of the products. Bismuth nanosheet structures possess large surface area brought about by the ultrathin structure, which can result in a substantially higher current density for the production of formate compared to bulk bismuth [11]. Moreover, bismuth metal is a poor electrocatalyst for hydrogen evolution reaction in an aqueous electrolyte system, which is advantageous for CO_2_RR [12].

Herein, electrodeposited bismuth on tin substrate (Bi/Sn) was prepared with different bismuth concentrations in the electrodeposition bath (0.001, 0.05, and 0.1 M) in order to investigate their effects on the bismuth morphology and electrochemical characteristics of the electrodes. The prepared Bi/Sn electrodes were employed in the CO_2_RR under the ternary mixture electrolyte system at the applied potential between −1.1 to −1.7 V vs. Ag/AgCl. The characteristics of solid carbon products formed during CO_2_RR over Bi/Sn electrocatalysts were investigated by means of various analytical techniques including Raman spectroscopy, scanning electron microscopy-energy dispersive X-ray spectroscopy (SEM-EDX), and transmission electron microscopy-energy dispersive X-ray spectroscopy-selected area electron diffraction (TEM-EDX-SAED).

## 2. Materials and Methods

### 2.1. Preparation of Bi/Sn Electrodes

Tin foils (99.998%, Alfa Aesar, Haverhill, MA, USA) having dimensions of 10 mm × 20 mm × 0.1 mm were used as the substrate. The substrates were abraded with 800 G sandpaper and washed with DI water. The working area of 10 mm × 10 mm was separated from the electrical contact area by a parafilm. The electrodeposition baths were prepared in different concentrations of bismuth nitrate pentahydrate (98%, Sigma-Aldrich, St. Louis, MO, USA) in 1 M nitric acid (Sigma-Aldrich). The platinum rod was used as an anode and tin foil was used as a cathode in a two-electrode system. The electrodeposition potential was controlled at −0.7 V for 600 s by the Metrohm Autolab Potentiostat. The electrodeposited electrodes in the deposition bath with bismuth concentrations 0.01 M, 0.05 M, and 0.1 M were named as 0.01Bi/Sn, 0.05Bi/Sn, and 0.1Bi/Sn electrodes, respectively. The electrodes were washed with DI water and dried at room temperature for 1 h before further investigation. 

### 2.2. Electrochemical Measurements and Electrocatalytic Tests

The H-cell type reactor was set up. The chamber contained 20 cm^3^ of catholyte and anolyte, which was separated by Nafion^®^ 117 membrane. The mixture of PC:[BMIM]BF_4_:water was used as catholyte in the volumetric ratio of approximately 7:4:1. The solution of 0.1 M KHCO_3_ was used as an anolyte. The prepared Bi/Sn electrodes were used as a working electrode (cathode) with Ag/AgCl reference electrode, while platinum foil (25 mm × 25 mm, 99.99%, Alfa Aesar, Haverhill, MA, USA) was used as a counter electrode (anode). The electrochemical impedance spectroscopy (EIS), was conducted with a frequency from 0.1 MHz to 0.01 Hz. Linear sweep voltammetry (LSV) was proceeded under CO_2_-saturated or N_2_-saturated electrolytes from –0.7 V to –2.0 V vs. Ag/AgCl with a scan rate of 10 mV/s. The electrocatalytic CO_2_RR performances of the prepared Bi/Sn electrodes were investigated in a CO_2_-saturated catholyte. Before the test, the catholyte was saturated by a CO_2_ (99.99%, Linde, Bangkok, Thailand) flow rate of 100 cm^3^/min for 1 h. During the CO_2_RR test, the CO_2_ flow rate was reduced to 20 cm^3^/min. The catalytic activity of electrodes was investigated at the applied potential range of −1.1 V to −1.7 V vs. Ag/AgCl for 70 min. 

### 2.3. Structure and Products Characterization

The synthesized Bi/Sn electrocatalysts and products were analyzed by several techniques. The electrodeposited catalyst morphology and surface composition were investigated by scanning electron spectroscopy (SEM) via Hitachi (S3400N, Tokyo, Japan) with the accelerating voltage of 15 kV and energy dispersive X-ray spectroscopy (EDX) via Apollo x (EDAX, Pleasanton, CA, USA) with Link Isis Series 300 program EDX, respectively. The surface metal oxidation state was analyzed by X-ray photoelectron spectroscopy (XPS, Kratos, Manchester, UK). The XPS spectra were obtained using the Amicus spectrometer (Kratos, Manchester, UK) with Mg Kα X-ray gun at accelerating voltage 10 kV and current of 20 mA. The elemental binding energy (Bi, Sn, C, O, and F) were investigated with reference to the C 1 s at 284.8 eV. The chamber pressure was less than 10^−5^ Pa.

The solid product from CO_2_RR on the electrode was characterized by a Raman spectrometer (Perkin Elmer Spectrum GX equipped using the UV line at 532 nm and a TE-cooled CCD detector; the laser output was 10 mW, Waltham, USA) and transmission electron microscope (TEM, JEOL (JEM-2010), Tokyo, Japan). The liquid products in the reactor were analyzed by the nuclear magnetic resonance (NMR, Bruker, MA, USA) technique on Bruker AV400 ultra shield 400 MHz with DMSO-d6 as the solvent. Gaseous products in the effluent were analyzed by gas chromatography with a thermal conductivity detector (GC-TCD, Shimadzu GC-2014, Kyoto, Japan).

## 3. Results and Discussion

### 3.1. Effect of Bi Concentration on Bi/Sn Electrode Morphology

**Figure 1:**
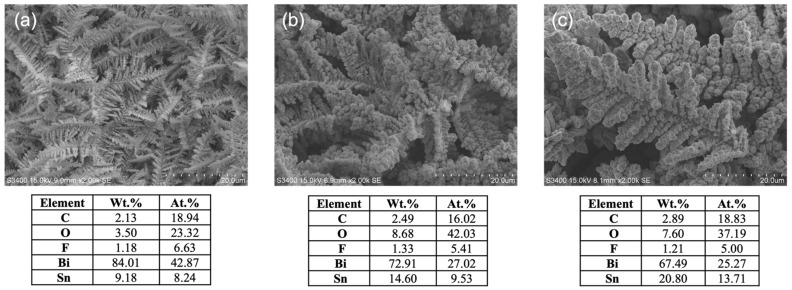
SEM-EDX results of (**a**) 0.01Bi/Sn, (**b**) 0.05Bi/Sn, and (**c**) 0.1Bi/Sn electrodes.

The SEM images of the prepared Bi/Sn electrocatalysts are shown in Figure 1. The morphology of bismuth electrocatalysts on tin substrate was dendrites for all Bi concentrations used in the electrodeposition bath. As the concentration of bismuth in the solution increased, the size of the dendrites became larger. Typically, dendritic structures are beneficial for catalytic reactions due to their larger surface area [13,14,15]. The smaller dendrite size with higher surface area could result in the improved electrocatalytic performances in CO_2_RR [16,17]. The EDX results showed that the Bi/Sn ratio on the electrodes decreased when the bismuth concentrations in the deposition bath increased. When bismuth concentration was lower (i.e., the 0.01Bi/Sn electrode), Bi atoms migrated near each other and spread throughout the surface in the atomic state to minimize the surface energy [18]. For the higher bismuth-concentration system, the number of reduced bismuth atoms was large; the nearest atoms caused them to clump together to create a massive nucleus. The size of each nucleus diffusion zone was determined by its size. The presence of trace F and C atoms on the electrodeposited catalyst surface was probably due to the impurities in the bismuth precursor. 

**Figure 2:**
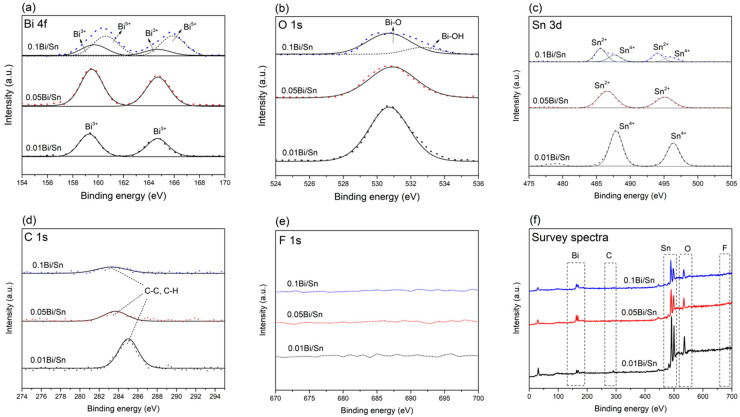
XPS spectra of (**a**) Bi 4f, (**b**) O 1s, (**c**) Sn 3d, (**d**) C 1s, (**e**) F 1s, and (**f**) survey spectra of Bi/Sn electrodes.

The XPS results of the Bi/Sn electrodes are shown in Figure 2. The Bi 4f peaks at 159 eV and 165 eV were observed on the Bi/Sn electrodes, indicating the Bi^3+^ on the surface as shown in Figure 2a. The presence of Bi^3+^ was mainly due to the exposure to air for 1 h before the CO_2_RR experiments [19]. A shift was observed from 159 eV and 165 eV to 160 and 166 eV for the 0.1Bi/Sn electrode as the Bi 4f peaks were slightly broadened [20]. The signal from oxygen species (O 1s) was observed at a binding energy of 531 eV, which was attributed to the Bi-O-Bi lattice bond (Figure 2b). A shoulder peak at 532 eV was observed on the 0.1Bi/Sn electrode, indicating the presence of the Bi-OH lattice bond on the surface [21]. Probably due to larger oxide film thickness on the 0.1Bi/Sn surface, it contained more Bi oxide species (Bi^3+^ and Bi^5+^). The tin substrate was also detected by XPS as an oxide species, Sn^2+^ and Sn^4+^ (Figure 2c), with the highest observed on the 0.01Bi/Sn electrode due to the incomplete coverage of bismuth on tin foil surface. The presence of carbon was also detected by XPS (Figure 2d). On the other hand, fluorine was not found in the XPS spectra (Figure 2e) of all electrodes due to the ultralow amount.

### 3.2. Electrochemical Measurement

**Figure 3:**
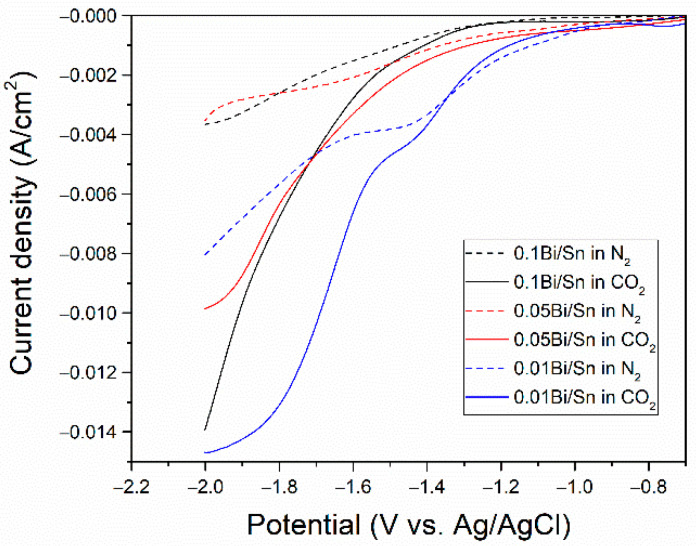
Linear sweep voltammetry (LSV) curves of the Bi/Sn electrodes in N_2_ (dashed line) and CO_2_-saturated (solid line) electrolyte.

To determine the applied potential in CO_2_RR, the linear sweep voltammetry (LSV) was carried out to compare the onset potential of hydrogen evolution reaction (HER) and CO_2_RR. In N_2_-saturated electrolytes (dashed line), the onset potentials were assigned to HER; in the CO_2_-saturated electrolyte (solid line), the onset shift could be assigned to CO_2_RR [22]. The LSV curves in Figure 3 revealed that the onset potential shifts for all the Bi/Sn electrodes in CO_2_-saturated electrolytes were in the range of −1.3 to −1.4 V vs. Ag/AgCl, suggesting that the size of bismuth dendrites on Bi/Sn electrodes did not affect the onset potential shift of CO_2_RR under our electrolyte system.

**Figure 4:**
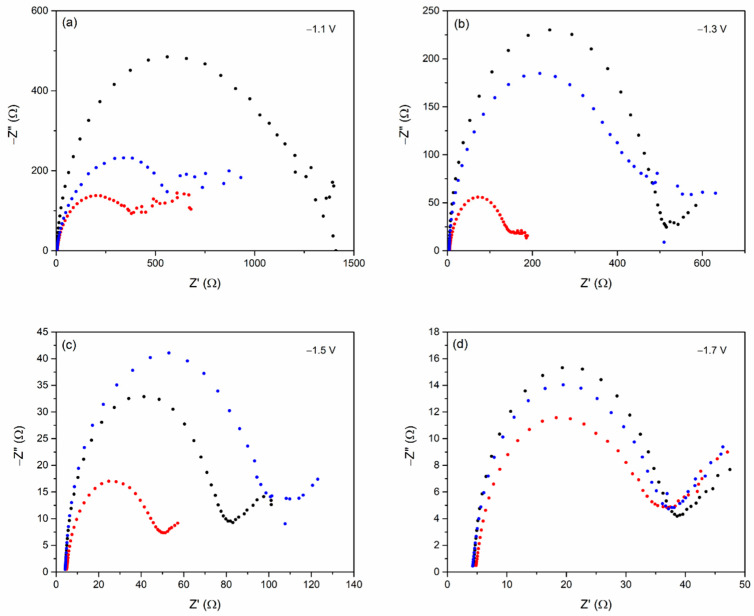
The Nyquist’s plot for 0.1Bi/Sn (black), 0.05Bi/Sn (red), and 0.01Bi/Sn (blue) electrodes at the applied potentials of (**a**) −1.1 V, (**b**) −1.3 V, (**c**) −1.5 V, and (**d**) −1.7 V vs. Ag/AgCl.

The electrochemical impedance spectroscopy (EIS) was used to investigate the charge transfer resistance (R_ct_) during the CO_2_RR. When the electrode was immersed in an electrolyte solution, an electrochemical double layer formed, with solvated ions forming a parallel plate that neutralized the electrode’s charge [23]. The dendritic size of bismuth on Bi/Sn electrodes can impact charge-transfer reaction and were reflected by charge-transfer resistance measured by EIS. The EIS results were presented in the Nyquist plots, as shown in Figure 4. The semicircle in the Nyquist plots indicate the charge transfer resistance [24], and the results are tabulated in Table 1. In this experiment, the Nyquist plots obtained at −1.1 V, −1.3 V, −1.5 V, and −1.7 V vs. Ag/AgCl showed the same tendency. The R_ct_ was found highest for the 0.01Bi/Sn electrode. The R_ct_ of the 0.05Bi/Sn electrode was significantly lower than the others under all conditions. It was probably due to the increase in the reactive area, including the edge site, by forming the porous structure [25]. Moreover, it was suggested that the increased potential was attributed to the decrease in R_ct_. In the CO_2_RR process, the lower the charge transfer resistance, the faster the charge transfer between the electron acceptor and the electrode, and the smaller the activation potential loss [26]. According to the results, the 0.05Bi/Sn electrode had the fastest charge transfer rate and the most favorable electrical transfer kinetics during CO_2_RR, which made it a promising effective electrocatalyst for CO_2_RR.

### 3.3. CO_2_RR toward Nanographene on the Bi/Sn Electrodes

**Figure 5:**
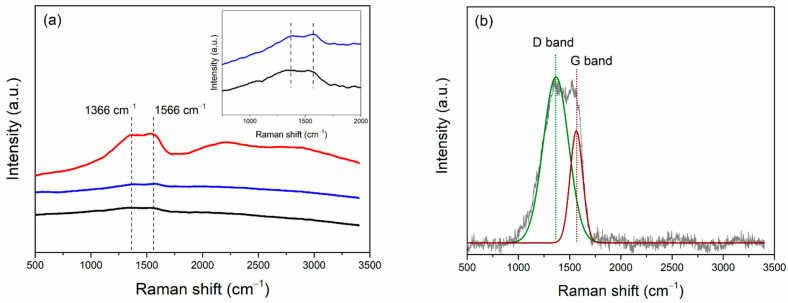
(**a**) Raman spectra of Bi/Sn electrode, 0.1Bi/Sn (black), 0.05Bi/Sn (red), and 0.01Bi/Sn (blue) and magnified scale (insert) of Raman spectra of 0.01Bi/Sn and 0.1Bi/Sn electrodes, and (**b**) Raman spectra analysis of 0.05Bi/Sn electrode after CO_2_RR test at −1.1 V vs. Ag/AgCl.

The fabricated Bi/Sn electrodes were tested for the performances in the CO_2_RR under the ternary mixture electrolyte system. The gaseous and liquid CO_2_RR products were analyzed by GC and NMR, respectively. After CO_2_RR for 70 min at the applied reaction potential −1.1 V vs. Ag/AgCl, there were negligible gaseous and liquid products according to the GC and NMR results (Figures S1–S4). Only solid carbon products were detected on all the prepared electrodes and were analyzed by Raman spectroscopy, TEM-EDX-SAED, and SEM-EDX. The Raman results in Figure 5a showed two peaks centered at around 1366 and 1566 cm^−1^, which is described as disordered, and graphite structure from the D and G bands around 1350 and 1580 cm^−1^ [4,27]. The Raman spectrum of 0.05Bi/Sn electrode was further analyzed and shown in Figure 5b. The high D band intensity indicates the high defect density [28]. The oxidation of graphene resulted in an increase in defect concentration, which was accompanied by a considerable broadening of D and G peaks in graphene oxide as compared to graphene [29]. Among the Bi/Sn electrodes prepared with different Bi concentrations in electrodeposition bath, the production of solid carbon products appeared to be better on the 0.05Bi/Sn electrode compared to the 0.001Bi/Sn and 0.01Bi/Sn.

**Figure 6:**
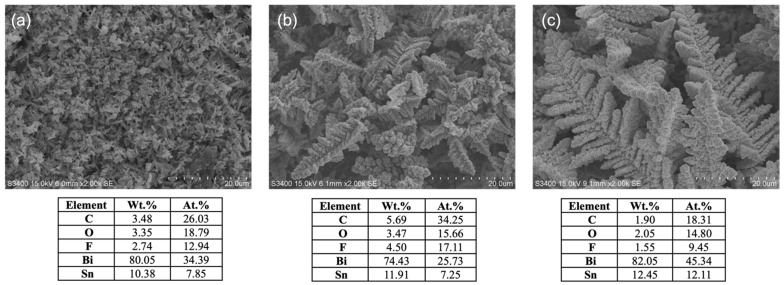
SEM-EDX results of (**a**) 0.01Bi/Sn, (**b**) 0.05Bi/Sn, and (**c**) 0.1Bi/Sn electrodes after CO_2_RR at −1.1 V vs. Ag/AgCl.

The SEM-EDX results of the tested electrodes are shown in Figure 6. No significant change was observed in the morphology of bismuth on 0.05Bi/Sn and 0.1Bi/Sn electrodes after the CO_2_RR test at −1.1 V for 70 min. The morphology of bismuth on 0.01Bi/Sn changed into a smaller size compared to the as-prepared ones due to the reconstruction process and the negative potential driving force during the reaction test [30]. It is suggested that the smaller size of Bi dendrites was less stable than the larger ones under the reaction conditions. The higher carbon percentages on the 0.05Bi/Sn electrode corresponded well with the higher carbon product formation. The EDX results reveal significant amounts of oxygen (37–45 at.%) on the electrode surface. These oxygens, either molecular or dissociative, can also be absorbed on Bi (111) as well as on the solid carbon products. They were not in the form of Bi_2_O_3_ because after reaction, only metallic Bi was detected. 

**Figure 7:**
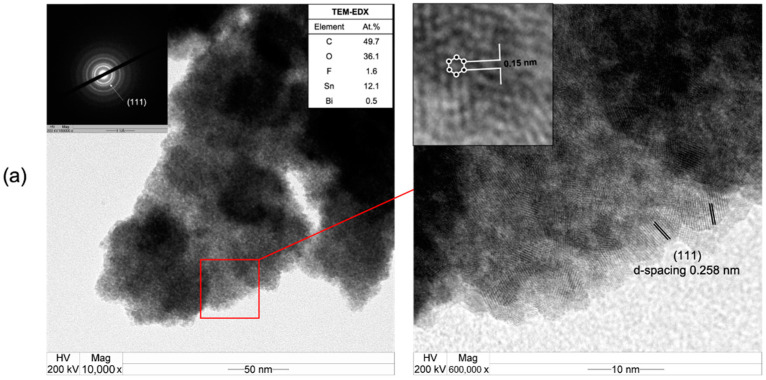

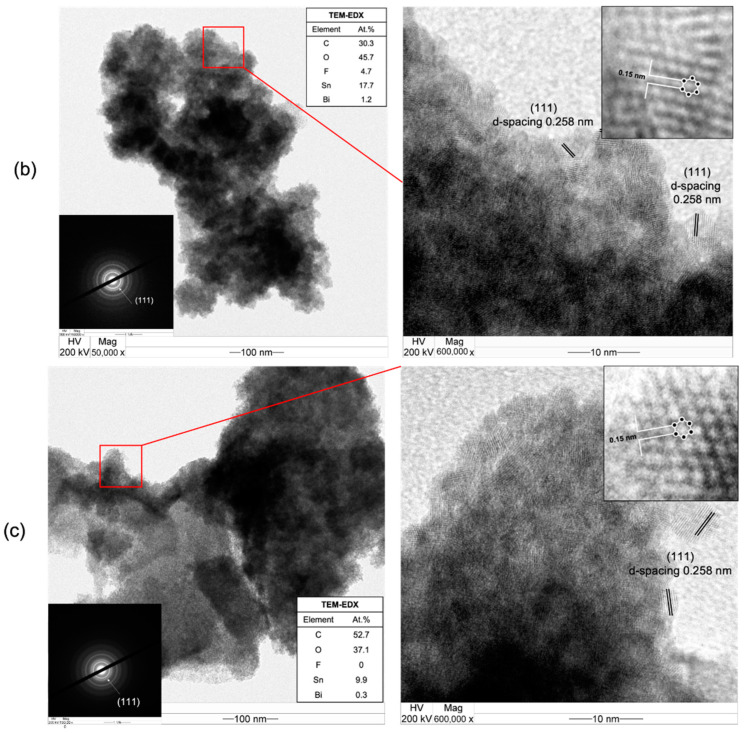
The TEM-SAED-EDX results of (**a**) 0.01Bi/Sn, (**b**) 0.05Bi/Sn, and (**c**) 0.1Bi/Sn electrodes after CO_2_RR test at −1.1 V vs. Ag/AgCl.

The TEM images in Figure 7 present the formation of polycrystalline nanographene with an average domain size of ca. 3–4 nm on all the Bi/Sn electrodes, regardless of the different morphology of the deposited Bi dendrites. The presence of a Bi (111) plane with a d-spacing of 0.258 nm was suggested by the corresponding SAED patterns. A hexagonal lattice structure with the C–C bond length around 0.15 nm is also evident. The crystallographic planes of the single-crystal metals generated by nanoclustering of the oxide layers of the bismuth metal electrocatalysts was completely aligned with the crystalline structure of the solid carbon products. The Bi (111) surface is the natural cleavage plane of Bi single crystals [31]. The growth of polycrystalline nanographene film occurs on the nascent Bi single crystals in situ formed upon nanoclustering of the Bi_2_O_3_. This could explain why different morphologies of the electrodeposited Bi/Sn yield similar structures of the nanocrystalline carbon products.

**Figure 8:**
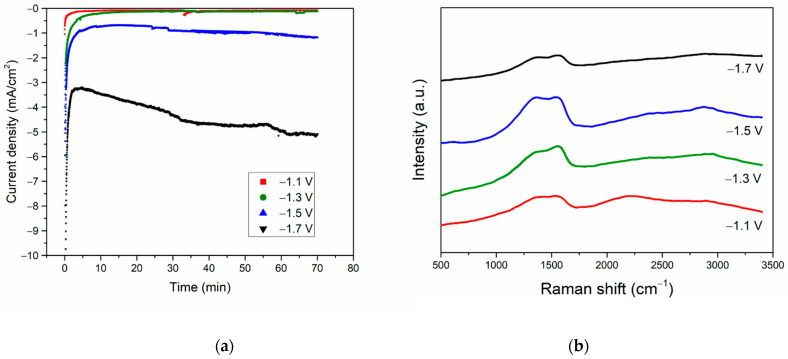
Current density of 0.05Bi/Sn during CO_2_RR at various applied potentials (**a**). Raman spectra of 0.05Bi/Sn electrode after CO_2_RR test at −1.1 V (red), −1.3 V (green), −1.5 V (blue), and −1.7 V (black) vs. Ag/AgCl (**b**).

Although the structure of solid carbon products formed was independent of the morphology of the electrodeposited Bi/Sn electrocatalysts, the carbon formation rate appears to be optimized on the Bi concentration 0.05 M and was found to increase with increasing negative values of the applied potential from −1.1 to −1.5 V vs. Ag/AgCl. The carbon formation was declined at the applied potential −1.7 V vs. Ag/AgCl. According to the current density plots shown in Figure 8a, the current density increased with increasing applied potential from −1.1 to −1.7 V. However, from the Raman spectra in Figure 8b, the highest intensity of the Raman signal was observed on the 0.05Bi/Sn electrode after CO_2_RR at −1.5 V vs. Ag/AgCl for 70 min and then declined for the one carried out at −1.7 V vs. Ag/AgCl. The gaseous products, CO and H_2_, were drastically increased at the higher negative applied potentials (Table 2). There were no liquid products formed under these conditions as determined from the NMR analysis (Figures S5–S7). 

The formation of polycrystalline nanographene on the electrodeposited Bi/Sn was a combination of both electrochemical and nonelectrochemical processes. Upon applying the negative potential, the stream of electrons moves toward the outermost surface of the electrodes where reduction of the ultrathin metal oxide layers (in this case Bi_2_O_3_) can generate the highly reactive negatively charged metal nanoclusters (NMCs). With excess high-energy electrons, the reduction of CO_2_ to CO and subsequent reduction of CO to C* atoms occur on the highly reactive NMCs, followed by the instantaneously C–C coupling into 3D-nanostructured carbon allotropes on the metal nanocrystalline facets as the template [3,4]. Only an initial start is required for the highly exothermic nonelectrochemical C–C coupling reaction, which leads to the growth of carbon allotropes. However, coalescence of the NMCs is a competitive pathway that can reduce surface energy of the NMCs. Agglomeration of NMCs would occur at a faster rate at higher negative applied potential, resulting in low activity for the reduction of CO_2_ to C and C–C coupling reaction. Therefore, at the highest applied potential, −1.7 V vs. Ag/AgCl, less-solid carbon products were formed, and the production of gaseous CO and H_2_ were significantly increased. The formation of solid carbon products was suggested to be optimized at around −1.3 to −1.5 V vs. Ag/AgCl using 0.05Bi/Sn electrode. Furthermore, the reaction became unstable due to gas bubbles formed on the electrode surface, especially from H_2_ generation under a higher negative potential [32,33]. The cross-sections of the 0.05Bi/Sn electrodes before and after CO_2_RR at −1.3 V vs. Ag/AgCl for 70 min are shown in Figure S8.

The power consumption and faradaic efficiency were determined at the applied potential of −1.5 V vs. Ag/AgCl according to the high intensity of Raman spectra and no hydrogen product. Under this condition, only solid carbon (defined as graphene) and CO product were detected. The power consumption and faradaic efficiency were calculated [34] and tabulated in Table 3. The primary calculated results showed that this process was profitable to produce graphene from CO_2_. On the other hand, the production of CO was not profitable at this electricity price. 

**Figure 9:**
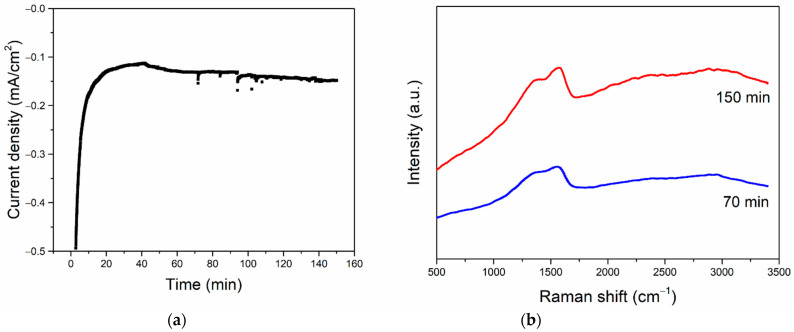
Current density of 0.05Bi/Sn electrode in CO_2_RR at −1.3 V vs. Ag/AgCl for 150 min (**a**) and Raman spectra of 0.05Bi/Sn electrode after CO_2_RR test at −1.3 V vs. Ag/AgCl for 70 min (blue) and 150 min (red) (**b**).

The stability tests were carried out on the 0.05Bi/Sn electrocatalysts at the potential of −1.3 V vs. Ag/AgCl. The current density was found to be fairly constant in the range of −0.1 to −0.2 mA during 150 min reaction time (Figure 9a). The formation of solid carbon products on the electrode during CO_2_RR under these conditions can possibly be enhanced by prolonging the reaction time from 70 min to 150 min (Figure 9b). 

### 3.4. Application of CO_2_-Derived Nanographene–Bi Composite in Electrochemical Sensor

**Figure 10:**
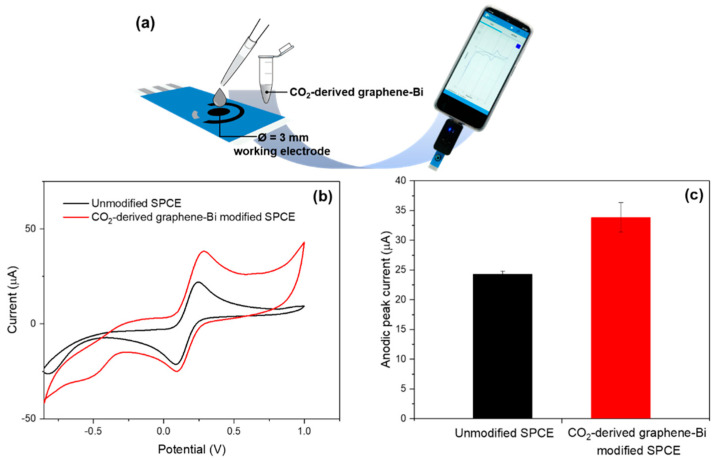
Illustration of CO_2_-derived graphene–Bi modified SPCE for electrochemical sensor application (**a**), cyclic voltammograms of 5.0 mM [Fe(CN)_6_]^3−/4−^ in 0.5 M KCl using unmodified and CO_2_-derived graphene–Bi modified SPCE (**b**), and anodic peak current obtained from the cyclic voltammograms in (**b**,**c**). The error bars correspond to the standard deviation obtained from three measurements (*n* = 3).

As for the potential application of using CO_2_-derived graphene–Bi composite as an additive in electrochemical sensors, the composite was dispersed in *N*,*N*-dimethylformamide by using ultrasonicator for 2 h, and 3 µL of the composite was directly dropped on the working electrode area of a screen-printed carbon electrode (SPCE), as shown in Figure 10a. By using cyclic voltammetry, the current response signal of a standard redox couple ferri/ferrocyanide ([Fe(CN)_6_]^3−/4−^) increased from 24 to 35 µA, which is approximately 1.5 times as shown in Figure 10b,c. These results verified that CO_2_-derived graphene–Bi composite enhances the electrochemical conductivity of the sensor, possibly due to the high conductivity of graphene and the high electrocatalytic property of Bi. Thus, this composite might be a potential additive for working electrode modification in electrochemical sensor-related applications. 

## 4. Conclusions

The Bi/Sn electrodes were prepared by electrodeposition of bismuth on Sn foil substrate with different bismuth concentrations in the electrodeposition bath (0.01 M, 0.05 M, and 0.1 M). After CO_2_RR at −1.1 V vs. Ag/AgCl for 70 min, solid carbon products were found on all the electrodes and were characterized as polycrystalline nanographene films with average domain size 3–4 nm. The morphology of the Bi dendritic particles did not affect the characteristics of the solid carbon formed, but higher activity was achieved on the 0.05Bi/Sn due probably to the lower charge transfer resistance and the higher stability of the electrodeposited Bi particles. The nanocrystalline carbon was grown on the nascent metal nanoclusters in situ formed by reduction and nanoclustering of the natural ultrathin oxide layers of the electrocatalyst during CO_2_RR. The optimized applied potential for solid carbon formation was determined to be around −1.3 to −1.5 V vs. Ag/AgCl using the 0.05Bi/Sn electrode. Increasing the negative value of the applied potential to −1.7 V vs. Ag/AgCl resulted in a slight deviation of product selectivity toward gaseous CO products and H_2_ evolution reaction. The current density of the electrocatalysts during CO_2_RR were stable during the 150 min reaction time. The results pave the way for a viable negative CO_2_ emission technology for CO_2_ utilization and a sustainable route for nanographene synthesis.

## Figures and Tables

**Table 1:** Charge-transfer resistance (R_ct_) of Bi/Sn electrodes analyzed by EIS.

Electrode	R_ct_ (Charge-Transfer Resistances) (Ω) at Each Potential (vs. Ag/AgCl)			
	−1.1 V	−1.3 V	−1.5 V	−1.7 V
0.01Bi/Sn	597.48	477.61	51.96	33.23
0.05Bi/Sn	392.77	144.40	46.04	31.89
0.1Bi/Sn	1015.44	516.42	78.68	34.41

**Table 2:** Gaseous products distribution detected by GC-TCD at various applied potentials.

Applied Potential (V) vs. Ag/AgCl	Production Rate (µmole/min)	
	H_2_	CO
−1.1	0.00	0.00
−1.3	0.00	0.01
−1.5	0.00	0.12
−1.7	2.87	0.47

**Table 3:** Faradaic efficiency, power consumption, and the market price of the products obtained from electrochemical reduction of CO_2_ on 0.05Bi/Sn at −1.5 V vs. Ag/AgCl.

Products	FE (%)	Input Energy (kWh/kg Product)	Energy Price * (Electricity) (USD/kg Product)	Product Price (USD/kg Product)
CO	21.4	8.22	0.82	0.6 [35]
C (graphene)	78.6	5.22	0.52	60–200 [36]

* Thailand electricity average price (USD 0.1/kWh).

## Data Availability

Not applicable.

## Supplementary Materials

The following supporting information can be downloaded at: https://www.mdpi.com/article/10.3390/nano12193389/s1, Figure S1: GC-TCD results of 0.01Bi/Sn, 0.05Bi/Sn, and 0.1Bi/Sn after CO_2_RR at −1.1 V vs. Ag/AgCl for 70 min; Figure S2: NMR spectra of catholyte results of 0.01Bi/Sn after CO_2_RR at −1.1 V vs. Ag/AgCl for 70 min; Figure S3: NMR spectra of catholyte results of 0.05Bi/Sn after CO_2_RR at −1.1 V vs. Ag/AgCl for 70 min; Figure S4: NMR spectra of catholyte results of 0.1Bi/Sn after CO_2_RR at −1.1 V vs. Ag/AgCl for 70 min; Figure S5: NMR spectra of catholyte results of 0.05Bi/Sn after CO_2_RR at −1.3 V vs. Ag/AgCl for 70 min; Figure S6: NMR spectra of catholyte results of 0.05Bi/Sn after CO_2_RR at −1.5 V vs. Ag/AgCl for 70 min; Figure S7: NMR spectra of catholyte results of 0.05Bi/Sn after CO_2_RR at −1.7 V vs. Ag/AgCl for 70 min; Figure S8: Current density of Bi/Sn during CO_2_RR at −1.1 V vs. Ag/AgCl for 70 min.
