# Supplementary material for: Room Temperature Nanographene Production via CO2 Electrochemical Reduction on the Electrodeposited Bi on Sn Substrate
Source: Nanomaterials (Basel). 2022 Sep 28;12(19):3389. doi: 10.3390/nano12193389 (PMC9565334; doi:10.3390/nano12193389)
Supplement: Supplementary file 1 [file nanomaterials-12-03389-s001.zip › nanomaterials-1926053-supplementary.pdf]

Supporting Information

Room Temperature Nanographene Production via CO_2 Electrochemical Reduction on the Electrodeposited Bi on Sn Substrate

Piriya Pinthong [1], Sarita Phupaichitkun [1], Suthasinee Watmanee [1], Rungkiat Nganglumpoon [1,2], Duangamol N. Tungasmita [3], Sukkaneste Tungasmita [2,4], Yuttanant Boonyongmaneerat [5], Nadtinan Promphet [5], Nadnudda Rodthongkum [5], and Joongjai Panpranot [1,2,6,*]

[1] Center of Excellence on Catalysis and Catalytic Reaction Engineering, Department of Chemical Engineering, Faculty of Engineering, Chulalongkorn University, Bangkok 10330, Thailand

[2] Graphene Electronics Research Unit, Faculty of Science, Chulalongkorn University, Bangkok 10330, Thailand

[3] Department of Chemistry, Faculty of Science, Chulalongkorn University, Bangkok 10330, Thailand

[4] Department of Physics, Faculty of Science, Chulalongkorn University, Bangkok 10330, Thailand.

[5] Metallurgy and Materials Science Research Institute (MMRI), Chulalongkorn University, Bangkok 10330, Thailand

[6] Bio-Circular-Green-economy Technology & Engineering Center, BCGeTEC, Department of Chemical Engineering, Faculty of Engineering, Chulalongkorn University, Bangkok 10330, Thailand

* Correspondence: joongjai.p@chula.ac.th

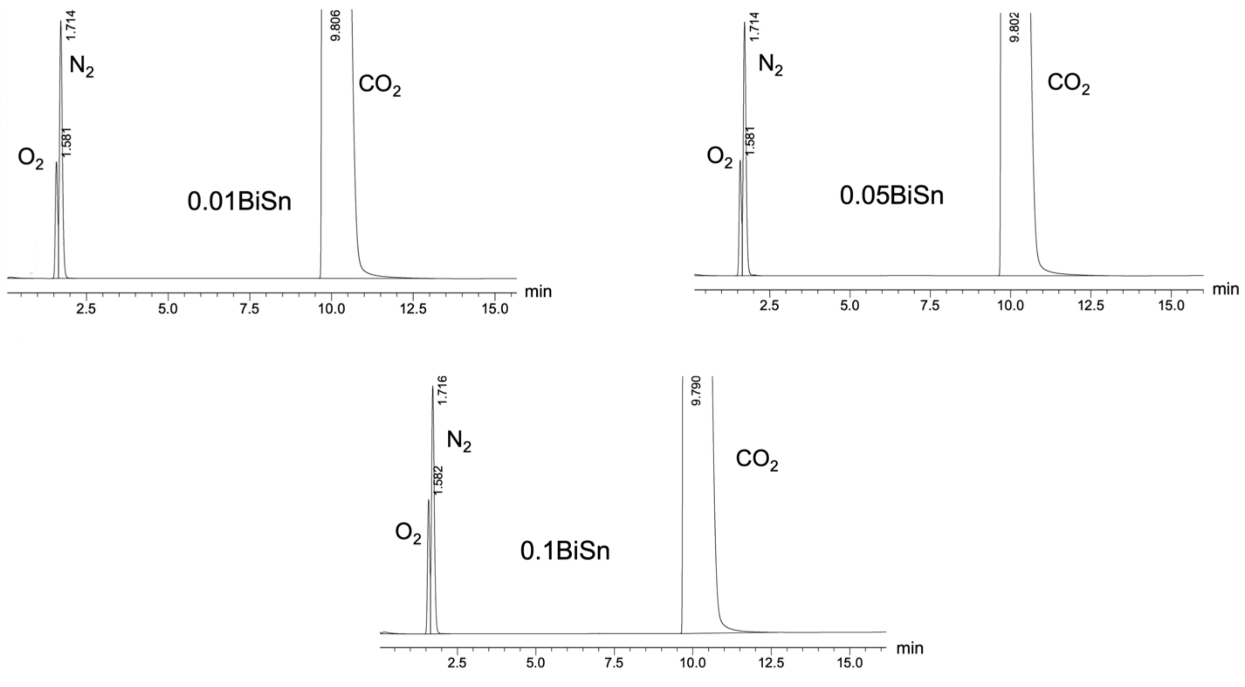

Figure S1 GC-TCD results of 0.01Bi/Sn, 0.05Bi/Sn, and 0.1Bi/Sn after CO_2RR at -1.1 V vs. Ag/AgCl for 70 min

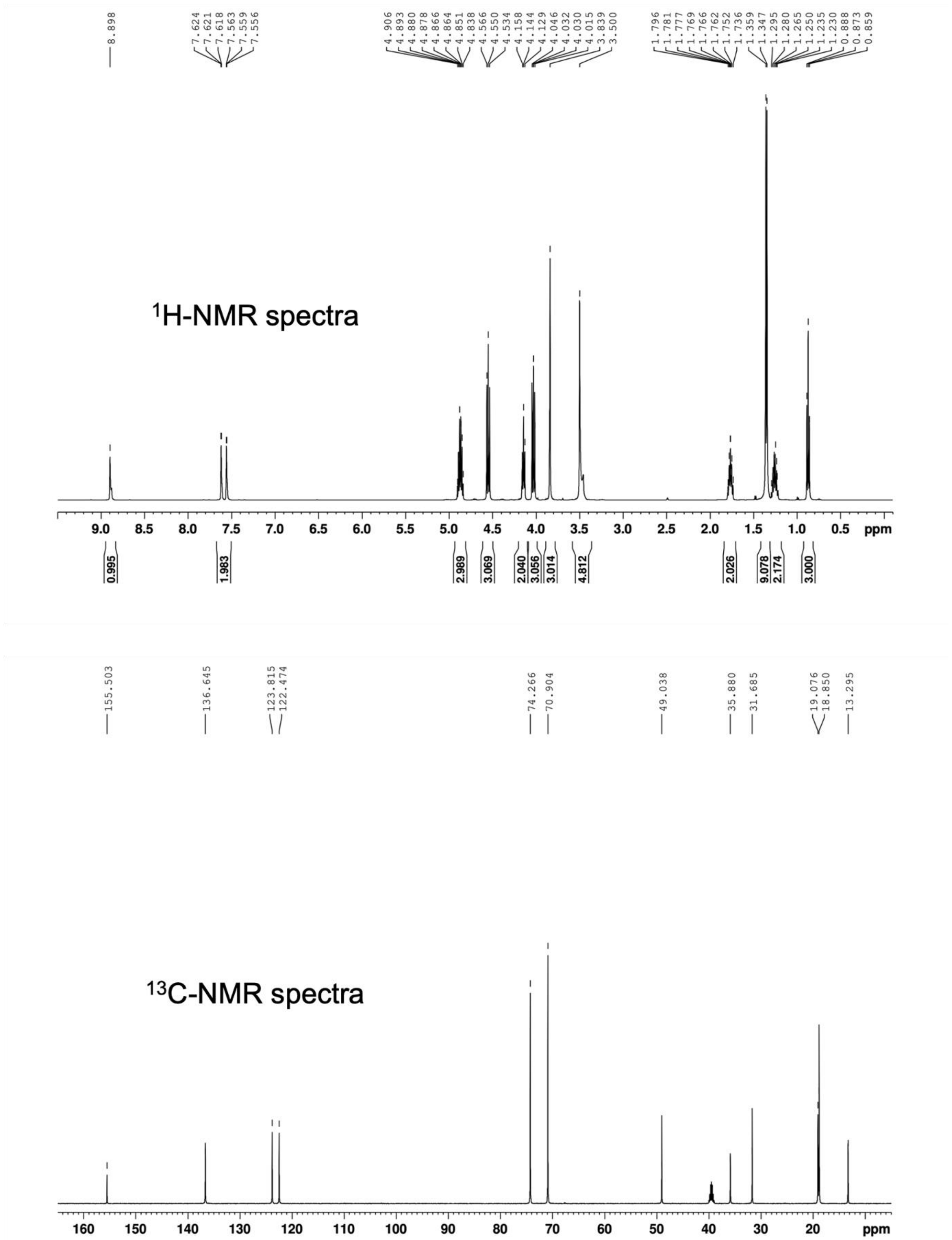

Figure S2 NMR spectra of catholyte results of 0.01Bi/Sn after CO_2RR at -1.1 V vs. Ag/AgCl for 70 min

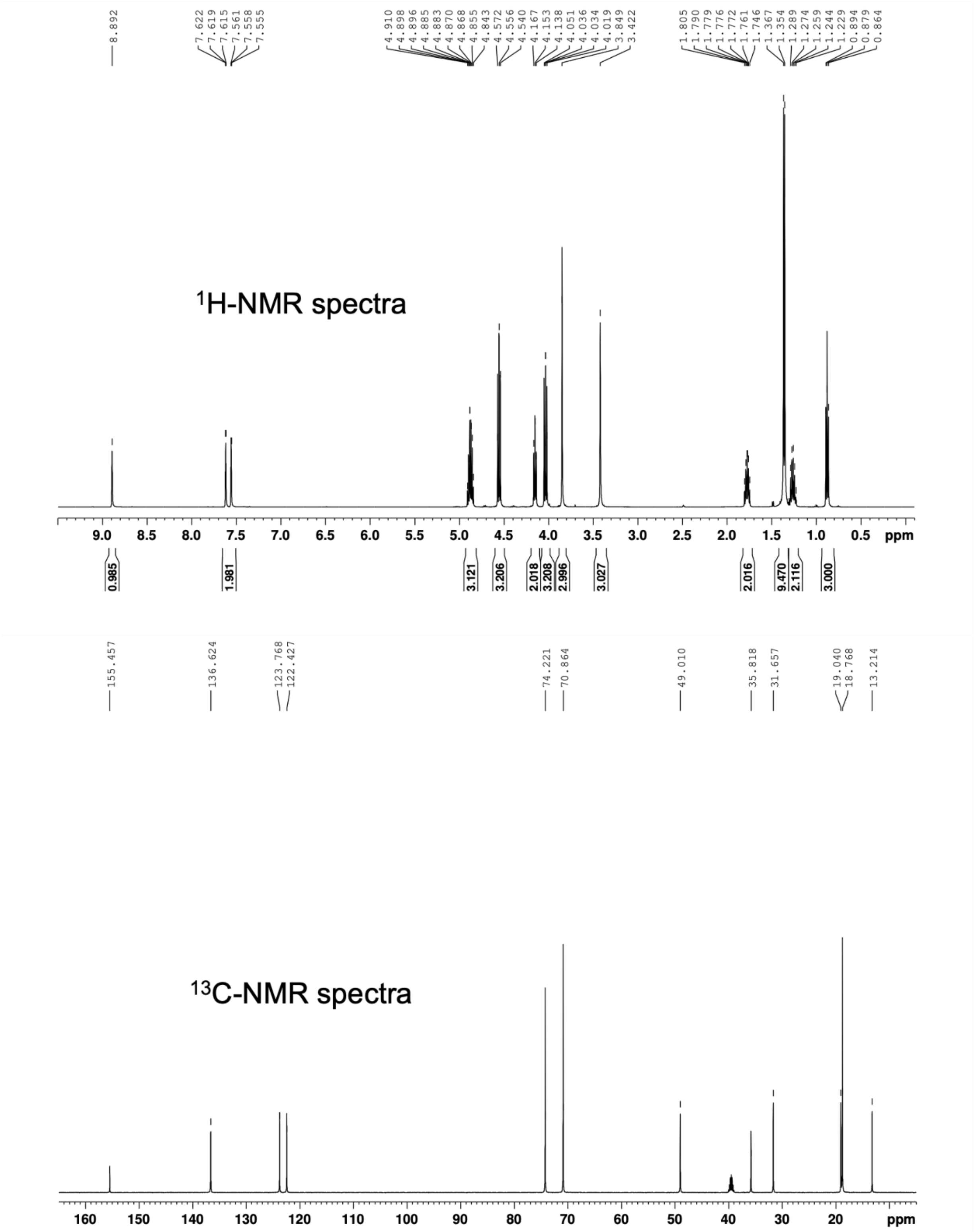

Figure S3 NMR spectra of catholyte results of 0.05Bi/Sn after CO_2RR at -1.1 V vs. Ag/AgCl for 70 min

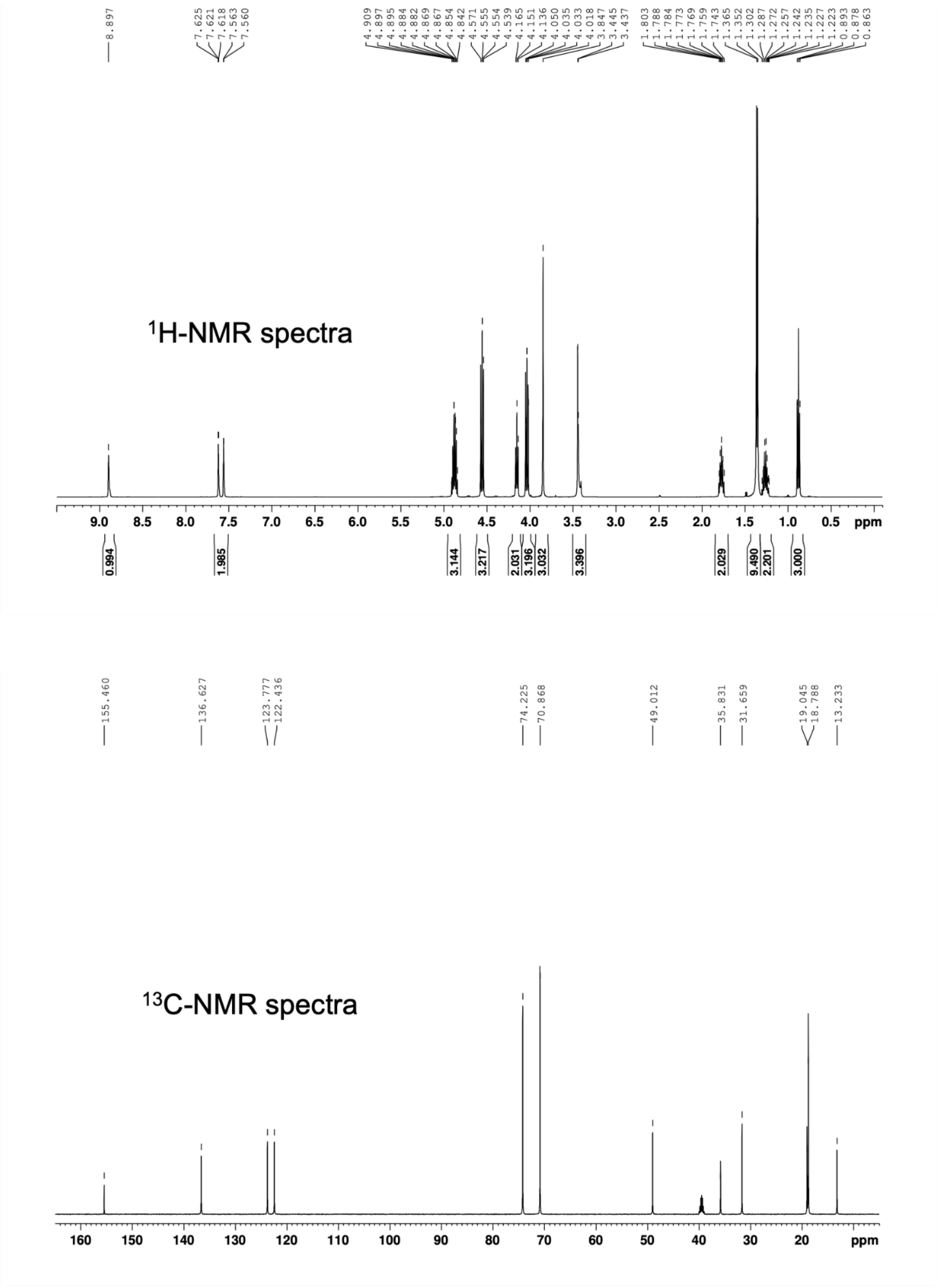

Figure S4 NMR spectra of catholyte results of 0.1Bi/Sn after CO_2RR at -1.1 V vs. Ag/AgCl for 70 min

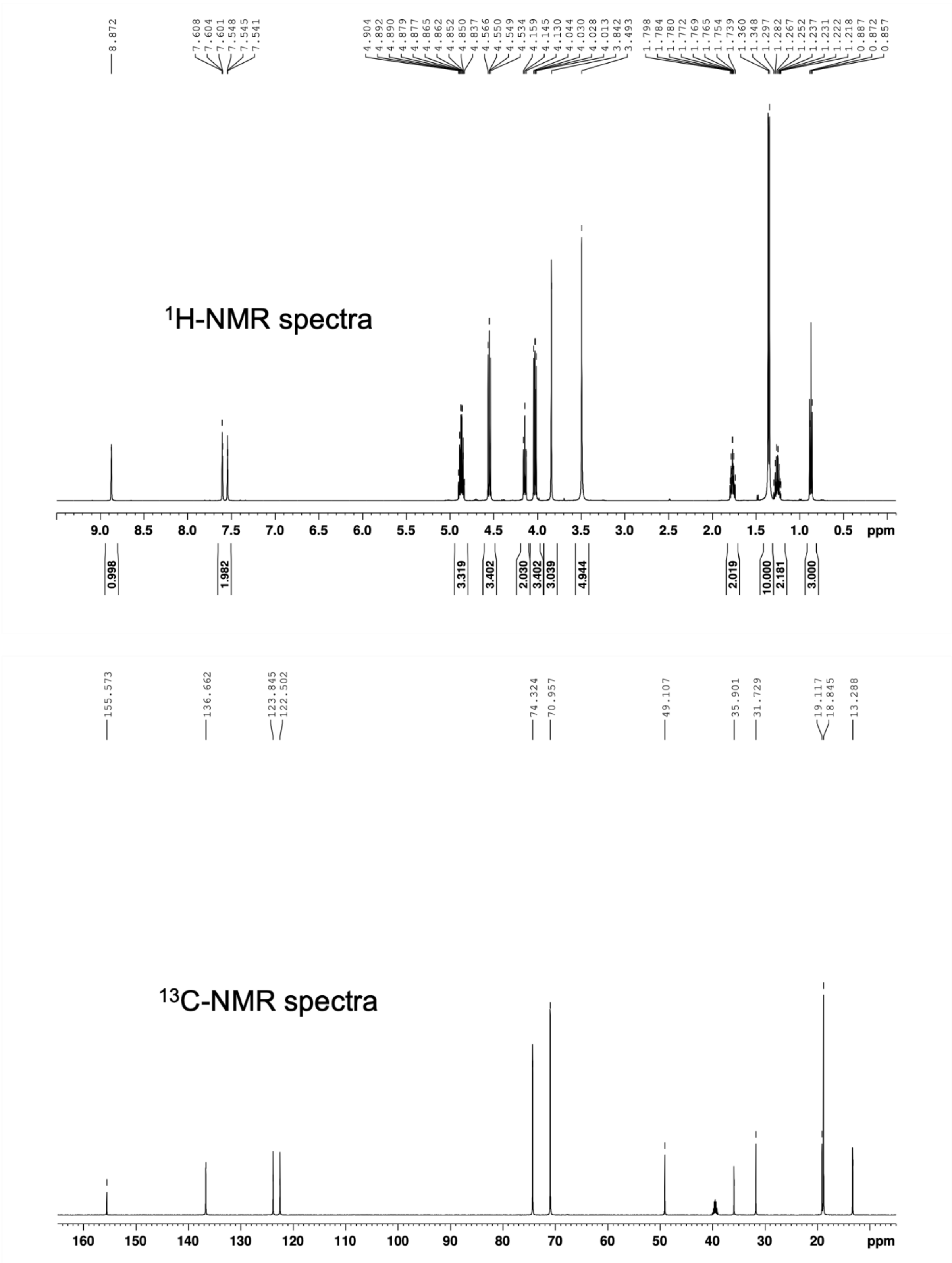

Figure S5 NMR spectra of catholyte results of 0.05Bi/Sn after CO_2RR at -1.3 V vs. Ag/AgCl for 70 min

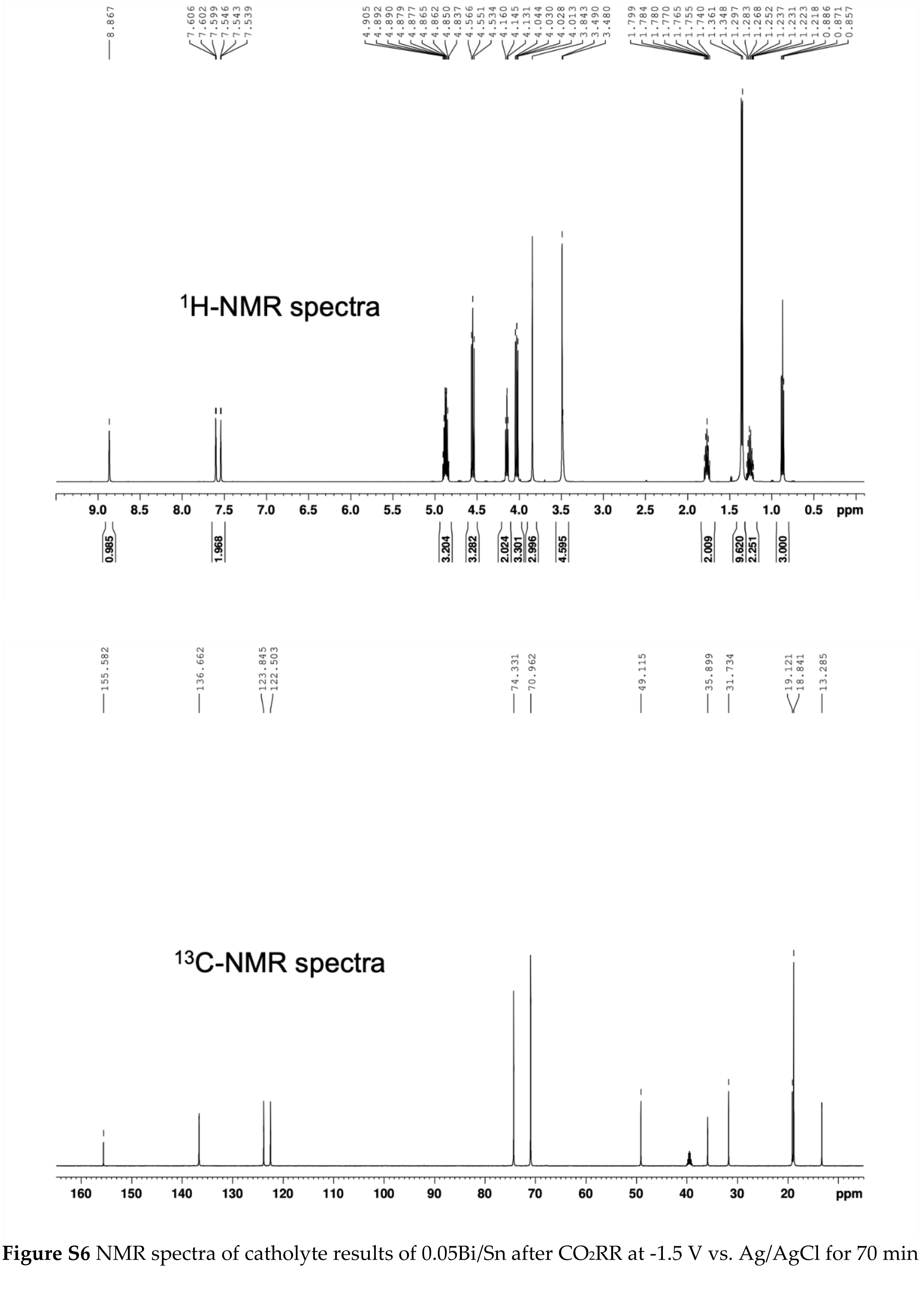

Figure S6 NMR spectra of catholyte results of 0.05Bi/Sn after CO_2RR at -1.5 V vs. Ag/AgCl for 70 min

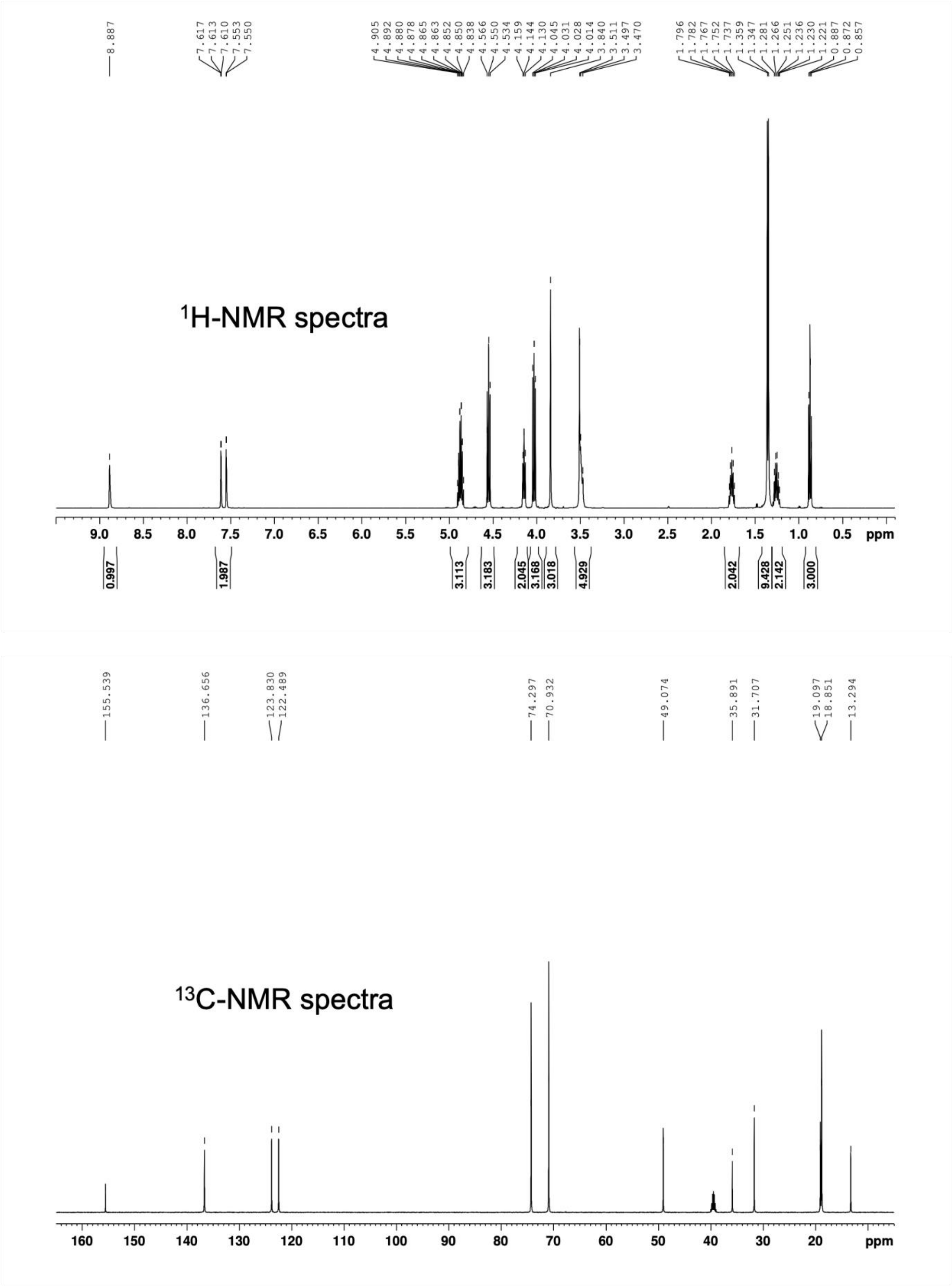

Figure S7 NMR spectra of catholyte results of 0.05Bi/Sn after CO_2RR at -1.7 V vs. Ag/AgCl for 70 min

(i) Analysis of ^{1}H-NMR spectra of cathodic electrolytes $[BMIM]^{+}[BF_4]^{-}$ and propylene carbonate before CO_2RR

Compounds	Molecular Structure	Chemical Shift (δ ppm)	
		Before Reaction (0 mins)	After Reaction (70 mins)
Propylene carbonate	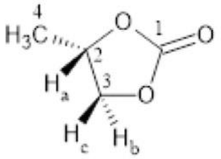	(H2a, 1H, t) 4.88	(H2a, 1H, t) 4.84
		(H3b, 1H, dd) 4.54, *J*=8 Hz	(H3b, 1H, dd) 4.52, *J*=8 Hz
		(H3c, 1H, dd) 4.04, *J*=8 Hz	(H3c, 1H, dd) 4.01, *J*=8 Hz
		(H4, 3H, s) 1.38	(H4, 3H, s) 1.35
1-Butyl-3-methylimidazolium tetrafluoroborate	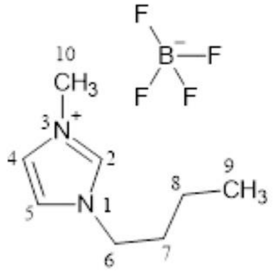	(H2, 1H, t) 8.96	(H2, 1H, t) 8.76
		(H5, 1H, t) 7.68	(H5, 1H, t) 7.53
		(H4, 1H, t) 7.61	(H4, 1H, t) 7.50
		(H6, 2H, t) 4.14	(H6, 2H, t) 4.09
		(H7, 2H, m) 1.72	(H7, 2H, m) 1.72
		(H8, 2H, m) 1.24	(H8, 2H, m) 1.29
		(H9, 3H, t) 0.87	(H9, 3H, t) 0.83
		(H10, 3H, s) 3.84	(H10, 3H, s) 3.84

(ii) Analysis of ^{13}C-NMR spectra of cathodic electrolytes $[BMIM]^+[BF_4]^-$ and propylene carbonate before CO_2RR

Compounds	Molecular Structure	Chemical Shift (ppm)	
		Before Reaction (0 mins)	After Reaction (70 mins)
Propylene carbonate	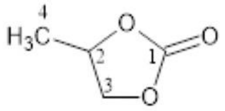	(C1) 155.37	(C1) 155.64
		(C2) 74.16	(C2) 74.33
		(C3) 70.88	(C3) 70.91
		(C4) 18.95	(C4) 18.80
1-Butyl-3-methylimidazolium tetrafluoroborate	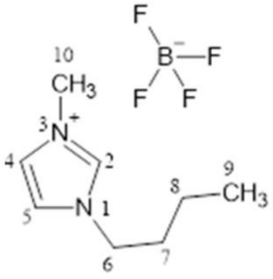	(C2) 136.80	(C2) 136.60
		(C4) 122.52	(C4) 122.30
		(C5) 123.84	(C5) 123.66
		(C6) 48.96	(C6) 49.09
		(C7) 31.73	(C7) 31.61
		(C8)19.28	(C8)19.07
		(C9)13.43	(C9)13.24
		(C10) 35.92	(C10) 35.70

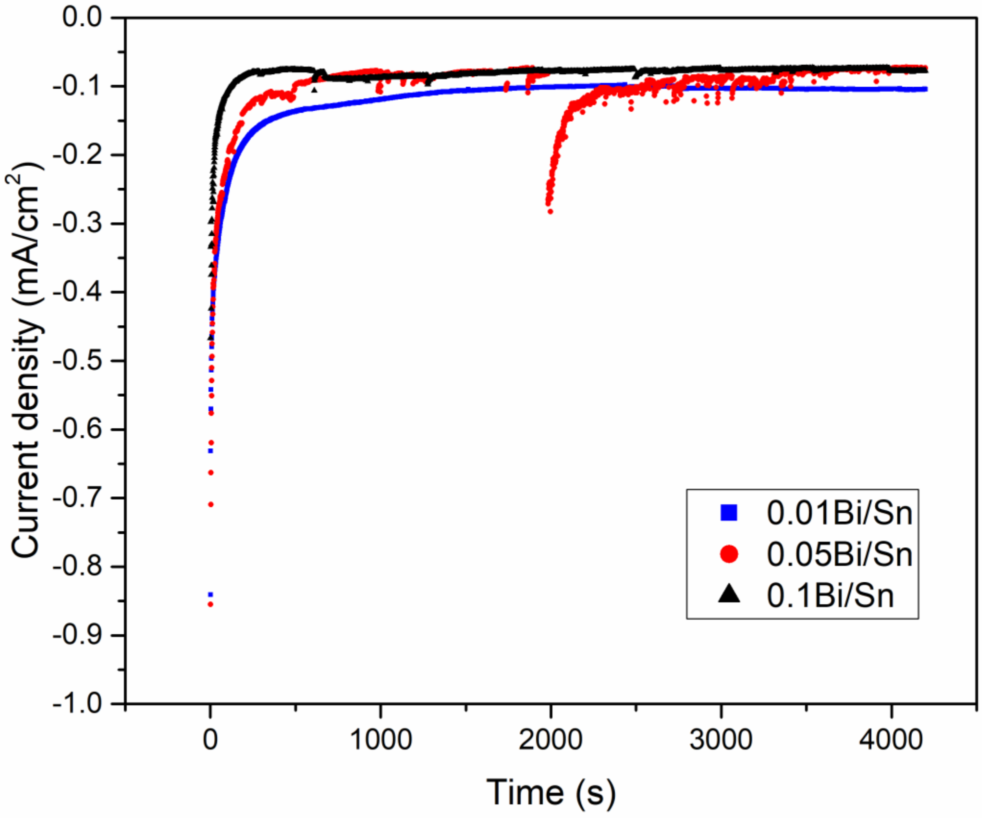

Figure S8 Current density of Bi/Sn during CO_2RR at -1.1 V vs. Ag/AgCl for 70 min

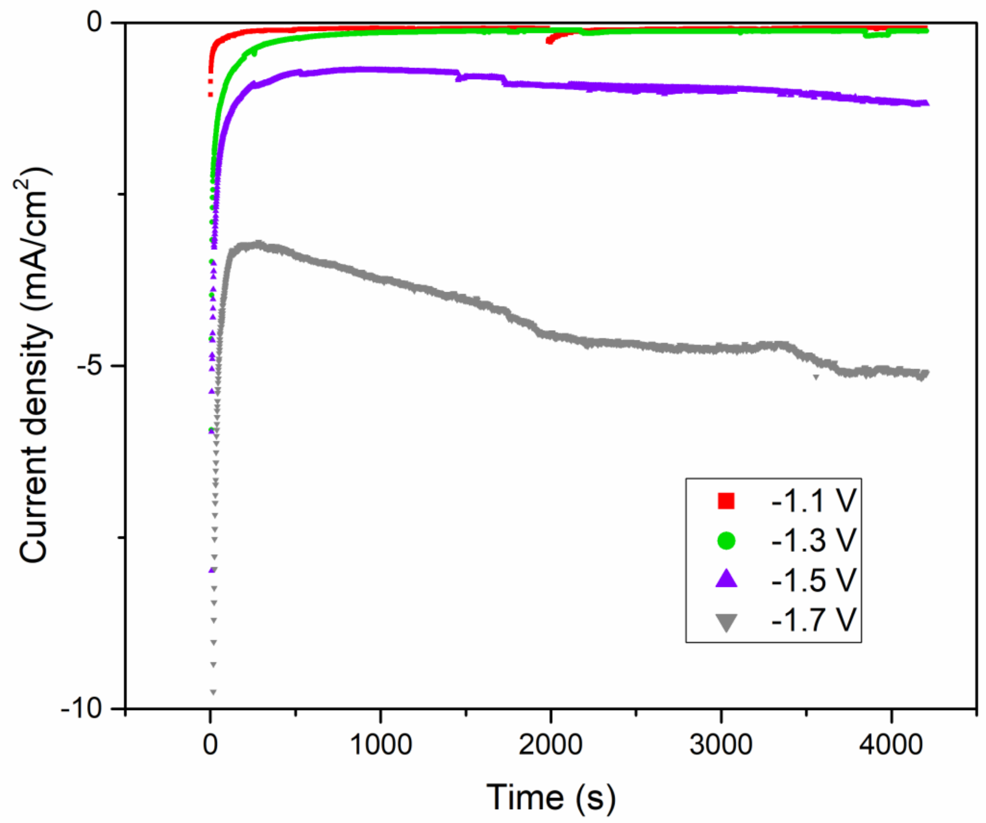

Figure S9 Current density of 0.05Bi/Sn during CO_2RR at various applied potentials vs. Ag/AgCl for 70 min
